# Selection of quality indicators for hospital-based emergency care in Denmark, informed by a modified-Delphi process

**DOI:** 10.1186/s13049-016-0203-x

**Published:** 2016-02-03

**Authors:** Michael Moesmann Madsen, Andreas Halgreen Eiset, Julie Mackenhauer, Annette Odby, Christian Fynbo Christiansen, Lisa Kurland, Hans Kirkegaard

**Affiliations:** Rigshospitalet, University of Copenhagen, Copenhagen, Denmark; Research Center for Emergency Medicine, University of Aarhus, Aarhus, Denmark; The Danish Clinical Registers, Aarhus, Denmark; Department of Clinical Epidemiology, Aarhus University Hospital, Aarhus, Denmark; Department of Clinical Research and Education, Karolinska Institutet, Stockholm, Sweden; Department of EM, Södersjukhuset, Stockholm, Sweden

**Keywords:** Emergency in-hospital care, Delphi, Delphi technique, ED, Emergency department, Emergency medicine, Key indicator, Performance indicator, Performance measure, Time-critical condition, Quality measure, Quality indicator

## Abstract

**Background:**

In 2013, Danish policy-makers on a nationwide level decided to set up a national quality of care database for hospital-based emergency care in Denmark including the selection of quality indicators. The aim of the study was to describe the Delphi process that contributed to the selection of quality indicators for a new national database of hospital-based emergency care in Denmark.

**Methods:**

The process comprised a literature review followed by a modified-Delphi survey process, involving a panel of 54 experts (senior clinicians, researchers and administrators from the emergency area and collaborating specialties). Based on the literature review, we identified 43 potential indicators, of which eight were time-critical conditions. We then consulted the Expert panel in two consecutive rounds. The Expert panel was asked to what extent each indicator would be a good measure of hospital-based emergency care in Denmark. In each round, the Expert panel participants scored each indicator on a Likert scale ranging from one (=disagree completely) through to six (=agree completely). Consensus for a quality indicator was reached if the median was greater than or equal to five (=agree). The Delphi process was followed by final selection by the steering group for the new database.

**Results:**

Following round two of the Expert panel, consensus was reached on 32 quality indicators, including three time-critical conditions. Subsequently, the database steering group chose a set of nine quality indicators for the initial version of the national database for hospital-based emergency care.

**Conclusions:**

The two-round modified Delphi process contributed to the selection of an initial set of nine quality indicators for a new a national database for hospital-based emergency care in Denmark. Final selection was made by the database steering group informed by the Delphi process.

**Electronic supplementary material:**

The online version of this article (doi:10.1186/s13049-016-0203-x) contains supplementary material, which is available to authorized users.

## Background

In recent years, hospital-based emergency care is undergoing major organisational change across several countries in Northern Europe. Several of the Nordic countries, as well as globally an increasing number of countries, are developing Emergency Medicine (EM) as a specialty as an answer to the increased demands on high quality EM care [1].

Concurrently, there is also a major drive internationally towards developing and refining quality indicators [2].

The setting of this study is hospital-based emergency care. Internationally, this corresponds to emergency department (ED) care, and it comprises the first part of the in-hospital patient management course. Typically, this includes triage, stabilization, initial diagnostics, work-up and preliminary treatment and visitation.

A 2007 report from the Danish Health and Medicines Authority mandated a nationwide re-organisation of hospital-based emergency care [3]. This reduced the number of departments receiving emergency patients from 45–50 to 21 newly established joint-specialty Emergency Departments.

The implementation of the novel EDs in terms of organizational set-up was to a large extent left open to each hospital and/or region, resulting in a wide range of different organisational set-ups [4]. In addition, a substantial share of emergency patient volume is still admitted directly to specialty wards [5].

In 2013, Danish policy-makers on a nationwide level decided to set up a national quality of care database for hospital-based emergency care in Denmark including the selection of quality indicators, which is the topic of this study. The database encompasses patient visits to the Emergency Departments as well as all unscheduled patient visits to all other in-hospital wards.

Concurrently with setting up the present database for hospital-based emergency care, two other databases, for pre-hospital care and trauma care, respectively, are being established. Altogether, these three databases have separate steering committees, but are jointly administrated as an umbrella-organization by the Danish Clinical Registers.

The aim of this study is to describe the Delphi process that contributed to the selection of quality indicators for a new national database of hospital-based emergency care in Denmark.

## Methods

Drawing on the structure-process-outcome triad described by Donabedian [6] as well as the framework by Institute of Medicine [7], we divide our potential indicators into the following categories: structure, process, outcome, equity as well as the time-critical conditions.

The Delphi method is a structured process for consensus-building among a diverse group of experts. The method was developed by the RAND Corporation in the 1950’ies [8], and remains today the most widely used method for selecting quality indicators in healthcare [9]. Specifically for selecting quality indicators for hospital-based emergency care, the RAND Delphi method has been used in many countries [9] including Canada [10], UK [11] and Ireland [12].

In this study, a process was conducted for identification of potential indicators followed by steering committee meetings for the selection of indicators. This process consisted in 1) a detailed literature review, and 2) a two-round Expert panel survey. This was followed by final selection of indicators by the database steering group.

Outside the scope of this selection process were a number of methodological considerations (e.g., case mix and organizational structure), which shall be included in the data set for description, stratification, adjustment and tabulation purposes. A nationally representative board committee (*N* = 14) developed and approved the methodology for the selection of quality indicators, determined the criteria for appointing members to an Expert panel (*N* = 54), and advised on dissemination of the results. Also outside the scope of the present project is the ongoing work to develop, refine and maintain the database. This work is expected to include further regular changes to the indicator set in coming years.

### Literature review and list of potential indicators

We reviewed the scientific literature to identify existing hospital-based emergency care quality indicators and time-critical conditions.

Initially, a gross-list of quality indicators and time-critical conditions was generated (see appendix) based on a literature review where review- and consensus papers from European and other western countries were given priority. The key sources were a systematic review of ED quality indicators [13], and a systematic review of the evidence for ED quality indicators [14]. Other sources were four consensus studies from Canada [10], the UK [11], Ireland [12] and South Africa [15] as well as quality measures identified by the Agency for Healthcare Research and Quality [16], and a report from the Danish think tank KORA on quality indicators in emergency departments [17].

Some indicators were *not* included in the gross list, despite favourable coverage in the international literature: Overall, indicators were assessed for relevance in a Danish context: For example, in Denmark, all patients have been seen or telephonically assessed in the primary sector before arrival at an ED, so *ambulance diversion* and related indicators are not directly relevant in a Danish setting. As another example, *patient satisfaction* is addressed in local, regional and national surveys, and is not part of clinical quality of care databases in Denmark. Patient satisfaction indicators were thus not included in our gross list. Furthermore, structural indicators, such as the number of care providers by type or the size of department (beds, patient throughput etc.) were not included as indicators, but some shall be part of the database as demographic/background variables, and were accordingly defined as beyond the scope of the present consensus process.

Next, the gross-list was reduced by the literature reviewer (JM) and the epidemiologist (CFC) to a net list of 43 potential indicators, of which eight were time-critical conditions. This step was taken based on a set of key considerations:

- The initial dataset should use only existing data (mainly the already mandatorily reported data to the national population-based registers) as clinicians should not be given any tasks for additional manual data registration of such a large population, neither could indicators be based on medical chart review
- Only readily available data could feed data for the initial database, i.e., data from ED logistic systems is still being processed, and is not available for all five regions in Denmark, but will deliver data for the next versions of the quality database
- Potential indicators were screened for feasibility, i.e., epidemiological operationalization: In Denmark, we have good quality clinical data available in a range of national databases. However, knowledge about strengths and limitation of potential data sources is required in order to judge whether data quality is or could be improved to a level sufficient for valid measurement of quality indicators. Our clinical epidemiologist on the team (CFC) judged each of our potential indicators on our gross list feasible for operationalization.

The 43 potential indicators / time-critical conditions were grouped based on the scope and source of each indicator, such that we differentiated between (i) global indicators, covering all unplanned hospital contacts (including emergency patients admitted directly to a ward, i.e., not through an ED), (ii) Tracer conditions, (iii) Emergency Department specific indicators, (iv) triage-specific, or (v) existing indicators from other national quality of care databases.

### Expert panel surveys

In selecting the Expert panel, care was taken to obtain a balance between the range of professional profiles, as well as a balanced regional representation across Denmark. In total, 55 experts were selected. The Expert panel included representatives from hospital administration, senior ED clinicians, ED organizational leaders, and professors of emergency medicine. The panel also represented 19 separate medical specialties, namely: eight internal medicine sub-specialties (cardiology, gastroenterology, rheumatology, pulmonary medicine, infectious disease medicine, nephrology, geriatrics and general internal medicine), four surgical specialties (general surgery, orthopedics, thoracic surgery and vascular surgery), two diagnostic specialties (radiology and clinical microbiology), neurology, family medicine, occupational medicine, anesthesiology and emergency medicine. Additionally, two registered nurses were in the panel. The experts received no incentives to participate.

The role of the Expert panel was to review the 43 potential indicators / time-critical conditions, provide comments and rate each with regards to its “usefulness as a quality indicator” for inclusion in the set of quality indicators. Participants were asked not to limit their views about the potential usefulness of an indicator by perceived difficulties in collecting or processing the data required to calculate it.

All 43 potential indicators / time-critical conditions were formatted into a questionnaire, to be completed and returned electronically. Individualized links to the survey were distributed by e-mail. We employed the online survey platform www.surveymonkey.com.

Before the two Expert panel rounds, and to ensure the quality of the questionnaire and comprehension of each question, we performed a pilot study of one round with three participants. This led to structural changes as well as added explanatory text. In each of the two Expert panel survey rounds, each indicator / time-critical condition was listed along with a short explanatory text. This was followed by the statement: “To what extent do you agree or disagree with: The above is a good indicator for the hospital-based emergency care database?” Answer options ranged discreetly from one (= disagree completely) through to six (= agree completely). An additional answer option for each was “I don’t know”. Associated with each indicator / time-critical condition was also a space for free-text participant comments. Round one allowed two weeks and round two allowed three weeks for responses. Follow-up on non-responders was done by email and telephone.

Between round one and two, data was collected and analyzed. A graph-based report was circulated to the experts with the panel distribution of scores (frequency count of answer choices), as well as median and average scores along with comments sorted by indicator. All free text comments were included verbatim in this report, and a summary of comments was provided up front for all views that were expressed by at least two participants. This report was distributed to all experts ahead of round two. Additionally, an individually tailored report with each panelist’s score in comparison to the group average and median for each indicator was provided directly to each panelist. This enabled each panelist to consider his or her own score in light of the group median, average and feedback from round one. Finally, based on the feedback by the Expert panel and before round two, several adjustments were made to the formulation of indicators and descriptions of these.

After round two, data was again analyzed and a summary report was generated. This report sorted the indicators by the median scores and provided a summary of participant comments. We had defined a median of five or above on the six-step Likert scale as consensus criterion for the Expert panel. A defined cut-off of the median Likert score is one of the most frequently used consensus criteria in Delphi processes for quality indicator selection [9]. The report clearly highlighted which indicators had achieved consensus in the Expert panel, and it constituted a basis for discussion by the steering group.

### Steering group selection of indicators

Decision on the final set of indicators and formulation of indicators for selected time-critical conditions was made via two in-person by the Steering group meetings following the two Delphi rounds.

The task of the steering group was to consider the objective of the process as a whole, namely to identify a set of indicators that were immediately implementable as well as fulfilling the attributes of good performance indicators. Additionally, the set had to be comprehensive and inclusive without exceeding the maximum number of ten indicators. The mandate of the steering group was to identify the best set of indicators overall, and they were free to include new indicators not previously part of the process.

### Blinding

The Expert panel surveys (first two rounds of the modified Delphi process) were conducted using a double-blinded design. The responses from the panel participants were blinded from one another, and the data analysis was conducted by two investigators, who were blinded from the identity of each respondent.

### Ethical considerations

Fifty-five potential Expert panel participants were asked to participate and had the opportunity to decline. Informed consent was included in the questionnaire. One expert declined to participate.

## Results

### Literature review and list of potential indicators

The literature review yielded 35 potential indicators and eight time critical conditions for review by the Expert panel. These are listed in Additional file 1: Table S1 (available online). The list of indicators included 23 (55.5 %) process, seven (15.9 %) outcome, three (6.8 %) equity and two (4.5 %) structural indicators as well as eight (18.2 %) time-critical conditions.

The 43 potential indicators/conditions encompassed four global indicators, eight time-critical conditions, six ED admission specific indicators, four triage specific indicators and 21 indicators from existing Danish quality of care databases.

### Expert panel surveys

We conducted two rounds of Expert panel surveys. We received questionnaires from 53 of 54 possible respondents in round one (96.3 %) and 48 of 54 possible respondents in Round two (88.9 %). In each round, all but one of the returned questionnaires were completed in full. All answers were included in the data analysis.

We included a set of questions about the respondent’s position, the results of which can be seen in Table 1. The majority 47 (86.6 %) of Expert panel participants were primarily involved with Emergency Medicine as clinicians 26 (48.1 %) or clinician-academics 21 (38.5 %). Only six participants (11.5 %) were primarily academics. More than two-thirds 38 (71.2 %) of the panel held managerial positions within the field, and about two-fifths 22 (41.2 %) held a position with economic/financial responsibilities. The Expert panel also included emergency system administrators and representatives from hospital administration, as well as senior clinicians from other emergency care specialties (i.e., cardiology, critical care/anesthesia and surgery),

**Table 1:** Delphi expert panel composition

		*n* (%)
Invitees		54 (100)
What are your primary involvement in the field of emergency/acute medicine	Clinical	25 (48,1)
	Academic	6 (11,5)
	Equally	20 (38,5)
	None of the above	1 (1,9)
	In total	52 (96,3)
Are you employed in a position with managerial responsibilities?	Yes	37 (71,2)
	No	15 (28,8)
	In total	52 (96,3)
Are you employed in a position with economical responsibilities?	Yes	21 (41,2)
	No	30 (58,8)
	In total	51 (94,4)
Where are your primary employment	Regional hospital	22 (42,3)
	University hospital	27 (51,9)
	Private	0 (0,0)
	Other	3 (5,8)
	In total	52 (96,3)
Complete answers		52 (96,3)

Self-reported categorizations by Delphi panelists in survey questionnaire included in Round 1 of the surveys

The results of the expert panel surveys are presented in Additional file 2: Table S2 (available online). In total, 32 of the 43 proposed indicators / time-critical conditions obtained a median of ≥ 5. Within each of the indicator groups the results were as follows:

(i). Global indicators: All four suggested indicators achieved a median of ≥ 5. From the qualitative comments the key concern of respondents was the need to adjust these for case-mix.
(ii). Time-critical conditions: Only three of eight suggested time-critical conditions achieved a median of ≥ 5. The overall critical comments on the time-critical conditions with regard to their suitability as indicators focused on the heterogeneity of the underlying conditions.
(iii). Emergency Department specific indicators: five of six suggested indicators achieved a median of ≥ 5 in this category. Comments focused on the need to adjust for case-mix and differences in local ED organization for these indicators.
(iv). Triage-specific: All four suggested indicators achieved a median score of ≥ 5. The comments from respondents indicate a significant variation in local use and application of Triage across Danish emergency departments.
(v). Indicators from existing databases: 18 of 22 suggested indicators achieved a median score of ≥ 5.

### Steering group selection of indicators

See Table 2 for the final set of indicators selected by the steering group.

**Table 2:** Final indicators chosen by the steering group, drawing on the Delphi process results and other sources

Original indicator no	Indicators adapted from Round 2	Final indicator no	Final indicator after Steering Group selection	Indicator type
1	Short-term mortality after arrival	1	Definition: Short-term mortality (7-days) after un-planned (emergency) attendance to hospital^a^	Outcome
3	Re-admission after completed acute process	2	Definition 72-h return rate after un-planned (emergency) attendance for short hospital courses (<24 h)^b^	Outcome
21	Time from arrival to triage	8	Definition^e^	Process
23	Rapid assessment and treatment of gastrointestinal bleeding: Circulatory impact	7	Definition (unchanged from existing database): Part (%) of patients with gastrointestinal bleeding stabilized within 60 min from arrival	Process
27	Symptoms of perforated abdominal organ: Time for surgery	5	Definition (unchanged from existing database): Part (%) of patients with perforated abdominal organ going to the operating theatre within 3 h from arrival	Process
New indicators selected by steering group				
	Time to treatment in stroke	3	Definition: Patients with acute ischemic stroke recieving trombolysis within 1 h from arrival^c^	Process
	Time to treatment for STEMI	4	Definition: Time from arrival to KAG for patients with STEMI upon arrival (median minutes with interquartile range (IQR))	Process
	Time to x-ray of forearm/wrist	6	Definition: Time from arrival to x-ray for patients with a request for x-ray of wrist/forearm (median minutes with interquartile range (IQR))	Process
	Time to bedside consultation	9a-b	Definition^e^	Process
			a)Timeliness of bedside consultation by a (any) doctor^d^	
			b)Timeliness of bedside consultation by a specialist doctor^d^	
Modified and subsequently discarded indicator from the Delphi process				
31	Hip fracture: Preoperative optimization: Patients seen by specialist surgeon 4 h from arrival for pre-operative assessment	Not included	Please see new indicator 9a) Time-to-doctor and 9b) Time-to-specialist	Process

^a^ all un-planned hospital contacts included (i.e., also emergency patients by-passing the ED), but through baseline data, able to adjust for admission place, time ect

^b^ all short (<24 h) hospital courses (un-planned/emergency contacts) are included, not only patients discharged from the emergency department

^c^ new indicator in the existing database – introduced in 2014(?)

^d^ Time-to-doctor and time-to-specialst can, based on baseline/background data, then be stratified to specific diagnosis, if requested

^e^ Still awaiting data availability before defining cut-off

Two of the generic outcome indicators regarding short-term mortality, and rate of 72 h returns were selected. This decision was consistent with the consensus reached on two outcome indicators the global indicator group (i) resp. the ED specific group (iii).

The Expert panel process reached consensus on three time-critical conditions: myocardial infarction (MI), appendicitis and ectopic pregnancy. For each of these time-critical conditions, a specific indicator was formulated by the steering group. In MI, the decision fell on new indicator for “Time from first hospital contact to coronary arteriography in patients with ST-elevation myocardial infarction (STEMI)”.

After consulting the existing databases, the steering group initially selected two indicators from the stroke-database and two indicators from each of the emergency surgery and hip fracture databases. These six indicators had all reached consensus in the Expert panel. The two emergency surgery indicators remained unchanged. Upon the second meeting, it was decided to expand one of the indicators from the hip fracture database (time to seen by surgeon/specialist doctor), to include time to first examination by a medical doctor for *all* patients attending an ED (i.e., not only patients with hip fractures, even though this patient group can be easily identified in this database).

Also, the steering group learned that the initially selected indicator “time to surgery” for patients with hip fracture was not an optimal indicator for evaluating service and efficiency within the patients’ first minutes/h of the in-hospital course, since hip fractures are not universally perceived as emergent conditions in Denmark, and are thus subject to in-hospital delay due to prioritization. Instead, it was decided to include a process indicator for “time to x-ray of the wrist”.

One of the indicators from the stroke database (time to CT scan) was discarded since it was deemed that the data granularity (day of CT, not time of day) was insufficient for this present database. Additionally, the steering group selected one indicator from an existing stroke database: Time to treatment for stroke: # of patients with acute ischaemic stroke for whom thrombolytic therapy is initiated within one hour of hospital arrival.

Finally, one triage indicator: “time to triage” was selected. There was broad agreement in the steering group that time measures are important. However, current data availability limitations regarding real-time data implied that time to triage is the only immediately implementable indicator in this category.

None of the equity indicators were selected; however there was a broad agreement in the steering group that it should be possible to measure equity across all indicators by analytical cross-section of the data set. Thus, it shall be possible to stratify the data by structural variables such as time of day, day of week or sex, age and gender. These variables, while not part of the selection process, will form part of the data set as background variables.

## Discussion

### Principal findings

This study has two key findings: First, this study describes the first application of the Delphi process for healthcare quality indicator selection in a Nordic context. In this regard, a Delphi Expert panel was consulted in two rounds, and 32 potential indicators / time-critical conditions were identified.

Subsequently, the steering group chose a set of nine indicators for initial implementation in the in-hospital emergency care quality database. These were: 1) Short-term mortality after arrival, 2) 72-h returns, 3) Timeliness of treatment for stroke, 4) Timeliness of treatment for STEMI, 5) Timeliness of surgery for patients having surgery during admission with suspected gastrointestinal perforation; 6) Timeliness of x-ray of the wrist, 7) Timeliness of hemodynamic stabilization of acute gastrointestinal bleeding, 8) Timeliness of triage, and 9) Timeliness of bedside consultation by a specialist.

Note that this is merely the initial set of indicators. The indicator set for this database is planned to be dynamic, and further revisions to the set of indicators are planned in coming years. We must also highlight that final operationalization was instituted after the selection of the indicators, and the explicit epidemiological definitions of the indicators are thus beyond the scope of this paper. These definitions can be found online [18].

### Comparability with other findings

The Delphi method has been used extensively to develop and/or identify quality indicators in healthcare [9]. Specifically in an ED setting, the Delphi method is also a widely used systematic approach for choosing quality indicators. We employed a two round, modified Delphi process – two rounds of Expert panel rating followed by two in-person meetings by the steering group. In a systematic review of Delphi method use for quality indicator selection [9], Boulkedid and colleagues included 80 studies. Sixtythree % used a *modified* Delphi method (i.e., a Delphi process that includes a physical meeting) and the median number of rounds was three, with a minimum of two and maximum of four rounds. In 35 (44 %) of the studies, a nominal review group met *after* the two Expert panel rounds. Specifically within ED quality indicator selection, a three round modified Delphi was used in all studies we found that employed the Delphi methodology. In this study, we employed a two-round Delphi process, followed by final selection by the steering group. Our steering group had a liberal mandate which included the ability to modify and/or introduce new indicators. This mandate is similar to that frequently held by nominal review groups in the literature [10]. Thus, while the steering group was not part of our Delphi process, they did hold an equivalent mandate to nominal review groups in other studies. Thus, in this regard, our methodology is broadly in line with common practice.

Our Expert panel composition encompassed an attempt to create a representative mix of senior emergency care clinicians, healthcare administrators, professors and policy makers. Previous studies have either used a similar mix [10, 11], or had an overweight of EM specialists and ED practitioners. We did not have any patient representatives in our expert panel. This can be considered a weakness of our approach, which we share with many other healthcare quality indicator selection processes. By including administrators as well as ED practitioners along with senior clinicians from other emergency care specialties (i.e., cardiology, critical care/anesthesia and surgery), it can be argued that our study involves a broader range of stakeholder perspectives than those relying predominantly on EM specialists and ED practitioners.

Our list of 43 potential indicators/conditions, with a distribution between *process* (54.5 %), *outcome* (15.9 %) and *equity* indicators (6.8 %) as well as time critical conditions (18.2 %), was roughly equivalent to the overall distribution between indicators in the emergency medicine indicator literature [9]. In our final set of nine indicators, seven were *process* and two were *outcome* indicators. This mix with predominantly *process* indicators is all but ubiquitous in ED quality indicator databases. The reason for the predominance of process indicators is likely *feasibility* constraints and *expedience* opportunities offered by ED logistics software and other in-hospital IT systems.

### Strengths and limitations

The literature review was selective and based largely on review articles [13, 14]. This implies that we have not been fully comprehensive and thus may have overlooked some internationally previously used potential indicators.

In the Expert panel rounds, we had a high response rate in both rounds, with 96.3 and 88.9% in round one and two, respectively. This is in the high end of response rates in this type of study, and should be considered a strength of our process since it implies we likely had a low non-response bias.

The final set of indicators have face validity in that they cover a range of the most serious health care emergencies seen in EDs, e.g., MI, and surgical emergencies. However, the external validity of the findings in this study may be limited because many of the agreed indicators probably reflect what the Delphi experts consider priorities in relation to ED quality improvement in Denmark. Other indicators, such as short-time mortality after arrival, time to triage and percent re-attendances within 72 h of discharge represent important indicators of ED efficiency and performance, which are of particular concern to healthcare administrators, policy makers, clinicians, and patients alike.

The steering group included a broad range of stakeholders, but some stakeholders may have been underrepresented. For example, ED nurses and primary care practitioners were not represented (as opposed to the Delphi Expert panel which included both of these stakeholder types). It is likely that different indicators will be deemed more or less relevant depending on the stakeholder audience; for instance, ED managers may prioritize different indicators than policy-level administrators.

Bias may have been introduced into the process in several ways. The process was very sensitive to how the questions were formulated. We took care to formulate the descriptions of each indicator in a neutral and unbiased way, however this type of bias is all but impossible to eliminate entirely. Overall, the time-critical conditions were rated lower than the other indicators. This could be because the underlying indicators were harder to imagine than those indicators that were more clearly and specifically articulated – a form of cognitive bias [19].

A commonly noted advantage of Delphi Expert panels is anonymity of participants, thus minimizing the risk that a strong group member could introduce bias by influencing the group. In the present process, the steering group met in-person for the final and decisive discussion and selection of indicators. There was no anonymous scoring of indicators by the steering group. Thus, there is a risk that this final step could have introduced this type of bias to the process.

It is a notable weakness of our study that we were mandated to consider for our gross list only indicators which could be operationalised based on existing nationwide data. This can have prevented potential innovative and very relevant indicators from being included. To this point, it is worth noting that the present set of nine indicators is to be updated in subsequent years, as more real-time national data becomes available.

This is the first time the Delphi method was used as part of the process to set up a clinical database in Denmark. Compared to the usual process for setting up a national clinical database in Denmark, where indicators are selected by a systematic literature review and a smaller specialty-specific Expert panel [20], the present study represented a substantially increased time-consumption. Additionally, the Delphi Expert panel rounds only led to a reduction from 43 to 32 indicators, and the steering group had the largest impact on indicator selection (Table 2). Prior to initiating the process, we had expected that round 2 would have been more decisive as to the final set of indicators, however this turned out to be limited by a) a relatively large share of indicators reaching consensus and b) that on more thorough epidemiological review several indicators were found to be too challenging to immediately implement and thus had to be discarded from the initial set of indicators.

However, the Delphi process did provide the additional benefit by pre-wiring a broad range of stakeholders who will be pivotal to successful implementation of the database. Given the wide range of stakeholders and high level of political interest in in-hospital emergency care, the additional stakeholder involvement may prove advantageous for implementation and use of this database going forward.

In a Nordic perspective, there is substantial current interest in quality indicator development in this field. Thus, not only the process but also the resulting indicators may prove of interest to policy makers, clinicians and researchers across the region.

## Conclusions

The Delphi process provided guidance by selecting a subset of indicators that are widely accepted as relevant by those who will drive improvement within hospital-based emergency care in Denmark. As an additional advantage compared to the usual way of setting up clinical databases in Denmark, the present process provided pre-wiring of a broad range of stake-holders who shall be crucial for the implementation and ongoing success of the database.

## Additional files

Additional file 1: Table S1. 43 potential indicators / time-critical conditions identified by literature review prior to Expert panel surveys. (DOCX 23 kb) Additional file 2: Table S2. Results of the expert panel surveys with aggregated responses from the Delphi Panel participants. (DOCX 19 kb)
