# Supplementary material for: Selection of quality indicators for hospital-based emergency care in Denmark, informed by a modified-Delphi process
Source: Scand J Trauma Resusc Emerg Med. 2016 Feb 3;24:11. doi: 10.1186/s13049-016-0203-x (PMC4739088; doi:10.1186/s13049-016-0203-x)
Supplement: Additional file 1: Table S1. — 43 potential indicators / time-critical conditions identified by literature review prior to Expert panel surveys. (DOCX 23 kb) [file 13049_2016_203_MOESM1_ESM.docx]

## Additional file 1: Table S1 – 43 potential indicators / time-critical conditions identified by literature review prior to Expert panel surveys

| **No.** | **Indicator** | **Indicator type** | **Indicator grouping** |
| --- | --- | --- | --- |
| 1 | Short-term mortality after arrival | Outcome | Global indicator |
| 2 | Short-term mortality after completed acute process | Outcome | Global indicator |
| 3 | Re-admission after completed acute process | Outcome | Global indicator |
| 4 | Variation of outcome indicators | Equity | Global indicator |
| 5 | Ankle injuries | Time-critical condition | Time-critical condition |
| 6 | Syncope | Time-critical condition | Time-critical condition |
| 7 | Mild head trauma /commotio | Time-critical condition | Time-critical condition |
| 8 | Geriatric multi-morbid patient | Time-critical condition | Time-critical condition |
| 9 | DZ03-diagnosis codes | Time-critical condition | Time-critical condition |
| 10 | Re-admission with critical diagnoses: Acute myocardial infarction (AMI) | Time-critical condition | Time-critical condition |
| 11 | Re-admission with critical diagnoses: Appendicitis | Time-critical condition | Time-critical condition |
| 12 | Re-admission with critical diagnoses: Ectopic pregnancy | Time-critical condition | Time-critical condition |
| 13 | Short-term mortality after arrival to emergency department | Outcome | ED specific |
| 14 | Short-term mortality after completed acute process in emergency department | Outcome | ED specific |
| 15 | Mortality for time-critical diagnoses in the acute sector | Outcome | ED specific |
| 16 | Re-admission after completed acute process in emergency department | Outcome | ED specific |
| 17 | Variation of outcome indicators in emergency department | Equity | ED specific |
| 18 | Admission rate | Structure | ED specific |
| 19 | Proportion of patients who are triaged at arrival | Process | Triage specific |
| 20 | Proportion of triaged patients that are seen by a doctor in timely manner | Process | Triage specific |
| 21 | Time from arrival to triage | Process | Triage specific |
| 22 | Temporal variation of triage-indicators | Equity | Triage specific |
| 23 | Rapid assessment and treatment of gastrointestinal bleeding. Indicator: Circulatory impact | Process | From existing database |
| 24 | Rapid assessment and treatment of gastrointestinal bleeding. Indicator: Time for endoscopy | Process | From existing database |
| 25 | Rapid assessment and treatment of gastrointestinal bleeding. Indicator: Direct transfer to endoscopy | Process | From existing database |
| 26 | Rapid assessment and treatment of gastrointestinal bleeding. Indicator: Restrictive blood component therapy | Process | From existing database |
| 27 | Symptoms of perforated abdominal organ. Indicator: Time for surgery | Process | From existing database |
| 28 | Symptoms of perforated abdominal organ. Indicator: Risk stratification | Process | From existing database |
| 29 | Symptoms of perforated abdominal organ. Indicator: Antibiotic Therapy | Process | From existing database |
| 30 | Symptoms of perforated abdominal organ. Indicator: Respiratory and haemodynamic optimization | Process | From existing database |
| 31 | Hip fracture. Indicator: Preoperative optimization | Process | From existing database |
| 32 | Hip fracture. Indicator: Surgical delay | Process | From existing database |
| 33 | Hip fracture. Indicator: Early mobilization | Process | From existing database |
| 34 | Hip fracture. Indicator: Basal mobility | Process | From existing database |
| 35 | Stroke. Indicator: time to hospitalization 1 (3 hours) | Process | From existing database |
| 36 | Stroke. Indicator: time to hospitalization 2 (4.5 hours) | Process | From existing database |
| 37 | Stroke. Indicator: time to hospitalization, transient ischemic attack (TIA) 1 (symptoms of TIA within the past 2 days) | Process | From existing database |
| 38 | Stroke. Indicator: time to hospitalization, transient ischemic attack (TIA) 2 (more than a TIA within the past month) | Process | From existing database |
| 39 | Stroke. Indicator: time to hospitalization, transient ischemic attack (TIA) 3 (single TIA more than two days ago) | Process | From existing database |
| 40 | Stroke. Indicator: Organisation of treatment and rehabilitation in a stroke unit | Structure | From existing database |
| 41 | Stroke. Indicator: Diagnosis by CT / MRI scan 1 (patients with acute stroke) | Process | From existing database |
| 42 | Stroke. Indicator: Diagnosis by CT / MRI scan 2 (patients with TIA) | Process | From existing database |
| 43 | Stroke. Indicator: Early mobilization | Process | From existing database |
