# Supplementary material for: Selection of quality indicators for hospital-based emergency care in Denmark, informed by a modified-Delphi process
Source: Scand J Trauma Resusc Emerg Med. 2016 Feb 3;24:11. doi: 10.1186/s13049-016-0203-x (PMC4739088; doi:10.1186/s13049-016-0203-x)
Supplement: Additional file 2: Table S2. — Results of the expert panel surveys with aggregated responses from the Delphi Panel participants. (DOCX 19 kb) [file 13049_2016_203_MOESM2_ESM.docx]

## Additional file 2: Table S2 - Results of the expert panel surveys with aggregated responses from the Delphi Panel participants

This table shows the aggregated responses from the Delphi Panel participants, summarized by median, minimum and maximum scores.

|  | Delphi Panel Round 1 | | | | Delphi panel Round 2 | | | | |
| --- | --- | --- | --- | --- | --- | --- | --- | --- | --- |
| Indicator/Time critical condition | Median | Min | Max | NAs | Median | Min | Max | Max | NAs |
| 1 | 5 | 1 | 6 | 1 | 5 | 2 | 6 | 6 | 1 |
| 2 | 5 | 2 | 6 | 0 | 5 | 2 | 6 | 6 | 1 |
| 3 | 5 | 1 | 6 | 0 | 5 | 2 | 6 | 6 | 2 |
| 4 | 5 | 1 | 6 | 1 | 5 | 1 | 6 | 6 | 0 |
| 5 | 4.5 | 1 | 6 | 3 | 4 | 1 | 6 | 6 | 1 |
| 6 | 5 | 1 | 6 | 3 | 4 | 1 | 6 | 6 | 1 |
| 7 | 4.5 | 1 | 6 | 3 | 4 | 1 | 6 | 6 | 1 |
| 8 | 4 | 1 | 6 | 6 | 4 | 1 | 6 | 6 | 5 |
| 9 | 4 | 1 | 6 | 6 | 3 | 1 | 6 | 6 | 3 |
| 10 | 5 | 1 | 6 | 2 | 5 | 1 | 6 | 6 | 0 |
| 11 | 4 | 1 | 6 | 1 | 5 | 2 | 6 | 6 | 2 |
| 12 | 5 | 1 | 6 | 9 | 5 | 1 | 6 | 6 | 3 |
| 13 | 5 | 1 | 6 | 5 | 5 | 2 | 6 | 6 | 0 |
| 14 | 5 | 2 | 6 | 3 | 5 | 1 | 6 | 6 | 0 |
| 15 | 5 | 2 | 6 | 2 | 5 | 2 | 6 | 6 | 0 |
| 16 | 5 | 1 | 6 | 2 | 5 | 2 | 6 | 6 | 0 |
| 17 | 5 | 2 | 6 | 3 | 5 | 2 | 6 | 6 | 0 |
| 18 | 4 | 1 | 6 | 1 | 4 | 1 | 6 | 6 | 5 |
| 19 | 5 | 1 | 6 | 1 | 5 | 1 | 6 | 6 | 0 |
| 20 | 6 | 1 | 6 | 1 | 5 | 3 | 6 | 6 | 0 |
| 21 | 5.5 | 1 | 6 | 1 | 5 | 2 | 6 | 6 | 0 |
| 22 | 6 | 1 | 6 | 4 | 5 | 2 | 6 | 6 | 0 |
| 23 | 5 | 1 | 6 | 6 | 5 | 1 | 6 | 6 | 3 |
| 24 | 5 | 1 | 6 | 7 | 5 | 1 | 6 | 6 | 4 |
| 25 | 4 | 1 | 6 | 9 | 4 | 1 | 6 | 6 | 4 |
| 26 | 4 | 1 | 6 | 8 | 5 | 1 | 6 | 6 | 7 |
| 27 | 5 | 1 | 6 | 6 | 5 | 2 | 6 | 6 | 4 |
| 28 | 4 | 1 | 6 | 23 | 4 | 1 | 6 | 6 | 11 |
| 29 | 5 | 1 | 6 | 10 | 5 | 2 | 6 | 6 | 6 |
| 30 | 5 | 1 | 6 | 9 | 5 | 1 | 6 | 6 | 4 |
| 31 | 5 | 1 | 6 | 8 | 5 | 2 | 6 | 6 | 4 |
| 32 | 5 | 1 | 6 | 6 | 6 | 2 | 6 | 6 | 5 |
| 33 | 5 | 1 | 6 | 8 | 5 | 1 | 6 | 6 | 7 |
| 34 | 4 | 1 | 6 | 25 | 4 | 1 | 6 | 6 | 13 |
| 35 | 5 | 1 | 6 | 8 | 5 | 1 | 6 | 6 | 4 |
| 36 | 5 | 1 | 6 | 10 | 5 | 1 | 6 | 6 | 5 |
| 37 | 4.5 | 1 | 6 | 15 | 5 | 1 | 6 | 6 | 10 |
| 38 | 4 | 1 | 6 | 14 | 4 | 1 | 6 | 6 | 8 |
| 39 | 4 | 1 | 6 | 16 | 4 | 1 | 6 | 6 | 12 |
| 40 | 5 | 1 | 6 | 9 | 5 | 1 | 6 | 6 | 9 |
| 41 | 6 | 1 | 6 | 6 | 6 | 1 | 6 | 6 | 5 |
| 42 | 5 | 1 | 6 | 12 | 5 | 1 | 6 | 6 | 11 |
| 43 | 5 | 1 | 6 | 13 | 5 | 1 | 6 | 6 | 12 |

NAs non-responders
